# Triple Enhancement for Sensitive Immunochromatographic Assay: A Case Study for Human Fatty Acid-Binding Protein Detection

**DOI:** 10.3390/bios12121166

**Published:** 2022-12-14

**Authors:** Nadezhda A. Taranova, Alisa A. Bulanaya, Anatoly V. Zherdev, Boris B. Dzantiev

**Affiliations:** A.N. Bach Institute of Biochemistry, Research Center of Biotechnology of the Russian Academy of Sciences, Leninsky Prospect 33, 119071 Moscow, Russia

**Keywords:** immunochromatography, gold nanoparticles, sensitivity enhancement, fatty acid binding protein, cardiomarker

## Abstract

The work considers a combination of three enhancing approaches for immunochromatographic assay (ICA) and the integration of their impacts into changes of the limit of detection (LOD). Human fatty acid binding protein (FABP), an early biomarker of acute myocardial infarction, was the target analyte. Starting from the common ICA protocol with an LOD equal to 11.2 ng/mL, three approaches were realized: (1) replacement of spherical gold nanoparticles with gold nanoflowers having a branched surface (20-fold lowering the LOD); (2) enhanced labeling of immune complexes via nanoparticle aggregates (15-fold lowering); (3) in-situ growth of bound nanoparticles by reduction of gold salts (3-fold lowering). Single and combined implementations of these approaches have been studied. It has been shown that the LOD decrease for combined approaches is close to the multiplied contribution of each of them. The final LOD for FABP was 0.05 ng/mL, which is 220 times lower than the LOD for the common ICA protocol. The efficiency of the enhanced ICA with three combined approaches was confirmed by testing human serum samples for FABP presence and content. The development presents a new efficient technique for rapid sensitive detection of FABP for medical diagnostics. Moreover, the demonstrated multiple enhancements could be applied for various demanded analytes.

## 1. Introduction

The modern practice of medical diagnostics, as well as safety control of consumer products and the environment requires easy-to-use and rapid analytical systems that allow for productive and widespread testing. Immunochromatographic assay (ICA) fully satisfies these requirements and, because of this, has been successfully introduced into analytical practice [1,2,3]. A test strip for ICA implementation contains all the necessary reagents in a dried form; it can be stored for a long time and directly used without any additional compounds or equipment. The assay is initiated by applying the sample to the test strip and does not require any further action from the user, who receives a visual result in 10–15 min.

However, to ensure this simplicity, analytical reactions are far from equilibrium, and the detected signal appears at the moment of immune binding. Therefore, the number of formed immune complexes is limited (it is less than in equilibrium systems), and the recorded signal is determined only by the amount and optical properties of the bound label without any possibility for increase, as regards enzyme labels. Due to these reasons, ICA is less sensitive, often significantly, than laboratory immunoassay techniques, such as microplate enzyme immunoassay. Therefore, new variants of ICA with higher sensitivity are in demand [4,5,6]. Such improved techniques make the detection of very low concentrations of analytes possible, thereby enabling the performance of point-of-care testing for compounds with low levels in tested samples. In addition, when using more sensitive tests, it is possible to dilute the tested samples significantly and, thereby, eliminate the possible negative influence of the matrix of these samples [7]. To date, a row of approaches to reach lower limits of detection (LOD) in ICA have been described [8,9,10]. However, a typical development in this field presents a new change and compares the achieved LOD with its value for the common ICA [11,12]. Combining several approaches in one system could potentially provide additional opportunities to achieve low LOD, but this strategy remains poorly characterized. In particular, it is still unclear to what extent the improvements achieved with different approaches can be multiplied.

Approaches for lowering the LOD in ICA can be divided into groups based on which components of the analytical system, and what processes in it, they act upon. Accordingly, of the greatest interest is the combination of approaches with different directions of their actions. Regarding such different and actively developing directions, the following can be indicated:

(1) the use of new nanodispersed markers, which are detected at lower concentrations and allow the immobilization of a larger number of receptor molecules [13,14];
(2) increased quantity of nanoparticles bound to the formed immune complex, by aggregation of different functionalized nanoparticles in the course of their movement to the binding zone and directly at this zone of test strip [15];
(3) growth of the detected analytical signal by increasing size of nanoparticle labels after their attaching to the binding zone of the test strip [6].

In our earlier developments of ICA for various analytes, the enhancing approaches corresponding to these three groups have been implemented and characterized, namely:

(1) the use of gold nanoparticles with branched surfaces, namely, gold nanoflowers (GNFs), instead of traditional spherical gold nanoparticles (sGNPs) [16];
(2) the use of several kinds of nanoparticles functionalized by a biotin–streptavidin interacting module for their aggregation in the course of ICA [17]; and
(3) in situ growth of bound gold nanoparticles by their catalysis of cationic gold reduction [18].

**Figure 1:**
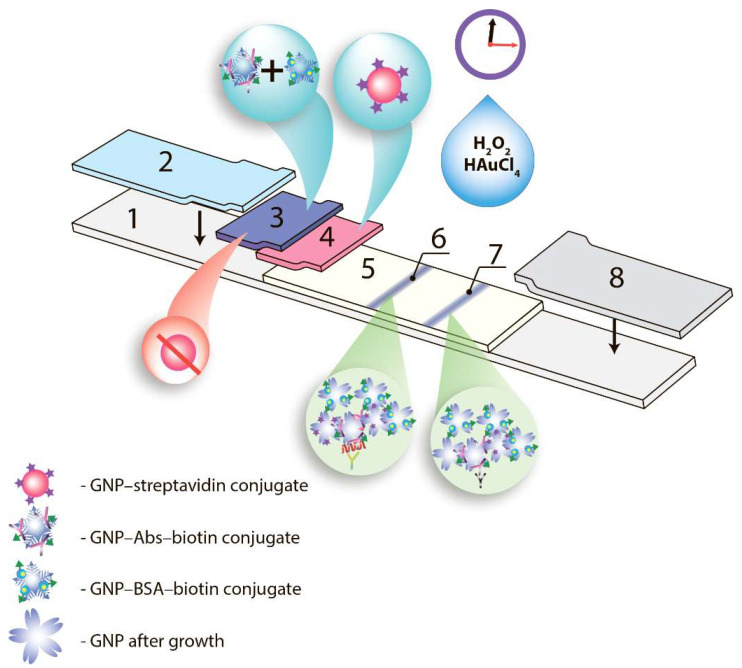
Scheme of test strip for ICA with triple enhancement: 1—plastic support, 2—sample pad, 3—pad with mixture of GNFs conjugates with biotinylated proteins, 4—pad with sGNP-streptavidin conjugate, 5—working membrane, 6—analytical zone, 7—control zone, 8—absorbent pad.

The given study was focused on the integration of these approaches in the same ICA system with an estimation of the achieved multiple enhancement, and the factors that influence the reaching of the low LOD. We started from the common ICA format, then considered and combined the three enhancing approaches named above (Figure 1).

The target analyte chosen for this study was cardiac isoform of human fatty acid-binding protein (FABP), a specific biomarker of cardiac muscle tissues that is released into the bloodstream when the cardiac muscles are destroyed. Cardiovascular diseases, and especially acute myocardial infarction, are important problems for healthcare, being the leading causes of death [19]. Among other cardiomarkers, the FABP stands out as the compound that most rapidly appears in the blood [20,21]. Therefore, tools for the sensitive and reliable detection of FABP are in demand. Their introduction in clinical practice provides information that can inform decisions about therapy for patients with cardiovascular diseases, heping to distinguish the causes of the observed symptoms [22,23]. After myocardial injury, FABP is released into the bloodstream within ~30 min, and is, therefore, useful for the early diagnosis of acute myocardial infarction, especially for patients with unstable angina [24,25].

## 2. Materials and Methods

### 2.1. Chemicals and Materials

Bialexa (Moscow, Russia) provided us with mouse monoclonal antibodies against human FABP (F5 and F10) and recombinant human FABP. The specificity of the antibodies was demonstrated in [26] by the absence of their cross-reactions with other blood components. The triple enhanced test system uses the same reactants. Goat anti-mouse IgG antibodies were purchased from Arista Biologicals (Allentown, PA, USA). Sigma (St. Louis, MO, USA) provided us with bovine serum albumin (BSA), streptavidin (Stp), biotinamidohexanoyl-6-aminohexanoic acid N-hydroxysuccinimide ester (biotin), Tween-20, Tween-80, Triton X-100, Pluronic 121 and sodium azide. Hydrogen tetrachloroayrate hydrate was purchased from Acros organics (Geel, Belgium). Other chemicals (such as solvents and salts) were of analytical (ACS) grade and were purchased from Chimmed (Moscow, Russia). Water for all solutions was purified by the Sartorius arium^®^ pro system (Sartorius, Göttingen, Germany) (18.2 MΩ cm). The immunochromatographic test system was made using Mdi Easypack (Advanced Microdevices, Gurudwara, India) membrane kits, used to fabricate a multi-membrane composite consisting of a working CNPC nitrocellulose membrane (15 µm pore size), PT-R7 glass fiber membrane and an AP045 adsorption membrane.

### 2.2. Synthesis of the Abs-Biotin and BSA-Biotin Derivatives

The Abs F10 and BSA were biotinylated at ratios of 15:1 and 8:1, respectively. The synthesis was described by Hermanson [27] and in our earlier publications [28]. During the synthesis, the biotin: Abs ratio was 15:1 and the biotin: BSA ratio was 8:1. Antibodies and BSA in PBS and biotin-NHS ester in dimethyl sulfoxide were mixed and incubated for 2 h at room temperature. Then, the reaction mixture was dialyzed against Tris-buffer (pH 9.0).

### 2.3. Preparation of sGNPs and Their Conjugates with Proteins

Spherical gold nanoparticles (sGNPs) with expected average diameters of 10 nm (as nuclei) and 30 nm were obtained by citrate reduction of HAuCl_4_ to Au^0^, as described by Frens [29]. Gold nanoflowers (GNFs) were obtained by subsequent growth of sGNPs, as described in [16], on the surface of which, gold salts were reduced using citrate and hydroquinone.

The GNPs conjugates with Stp, BSA-biotin, F10 and F10-biotin were obtained through physical adsorption [28]. BSA was used at a concentration of 0.04 mM to stabilize the gold nanoparticles (excluding their aggregation during storage), as recommended in [28]. After centrifugation of the synthesized conjugates, the precipitate was redissolved and stored at 4 °C.

### 2.4. GNPs Characterization

To carry out transmission electron microscopy (TEM), GNPs were applied to 300-mesh grids (Pelco International; Redding, CA, USA) pre-treatment with a poly(vinyl formal) film. Then, the film was placed on the glass and exposed to 0.15% v/v solution of formvar in chloroform [16]. The JEM CX-100 microscope (Jeol; Tokyo, Japan) was used to take images at 80 kV, with further processing by the Image Tool software (University of Texas Health Science Center, San Antonio, TX, USA).

Zetasizer Nano (Malvern Panalytical; Malvern, UK) was used for measuring the hydrodynamic size and zeta-potential GNPs; the dynamic light scattering of the nanoparticles was registered at a scattering angle of 173° for 10 s at 25 °C.

Biochrom Libra S80 spectrophotometer (Biochrom; Cambridge, UK) used to get the absorption spectra of the GNPs in the range of 350–800 nm.

For scanning electron microscopy (SEM) of the GNPs at the nitrocellulose membrane, the Tescan Mira 3 (Tescan, Brno, Czech) microscope with the AZtecOne X-act system for energy dispersive analysis (and a Schottky cathode Oxford Instruments, Abingdon, UK) was applied. The samples were pre-treated with carbon using the Q150R ES Plus spraying device (Quorum Technologies, Lewes, UK).

### 2.5. Fabrication of Immunochromatographic Tests

Assembly of immunochromatographic test strips was carried out as described in [17]. For the common test system, the GNPs conjugates optical density was 2.0; for the triple test system, the optical densities of the GNP–F10–biotin and GNP–BSA–biotin were 2.0 and 4.0, respectively, and the optical density of the GNP–Stp conjugate was 0.5.

IsoFlow dispenser (Imagene Technology, Hanover, NH, USA) was used to form the analytical zone (F5, 2 mg/mL, PBS) and the control zone (goat anti-mouse IgG, 1 mg/mL, PBS) onto the working membrane. The protein deposition rate was 1.2 µL per cm of membrane length, with further drying at room temperature for at least 20 h [30].

The multimembrane composites were assembled, and the sheets were cut into 3.9 mm-wide strips and stored at room temperature with relative humidity under 30% [31].

### 2.6. Immunochromatographic Assay and Data Processing

TruLab N serum (DiaSys Diagnostic Systems, Holzheim, Germany) was used to prepare the stock and working antigen solutions. The procedure for carrying out the ICA with the assembled test strips and their subsequent quantitative processing is described in [28]. The test post-assay enhancement of coloration was realized after the implementation of the common ICA. A mixed water solution containing 1.8% H_2_O_2_ and 0.085% HAuCl_4_ was prepared for the enhancement. 10 μL of this solution was applied to the test zone of the horizontally located test strips by dropping from an automatic pipette. The test strips were incubated for 1 min at room temperature and then their images after the enhancement were registered. To characterize the errors, the number of ICA repetitions was at least 5. The CanonScan 8800F Photo scanner (Canon, Suwa, Japan) and TotalLAB software (Nonlinear Dynamics, Newcastle, UK) were used to obtained the quantitative results.

The visual limit of detection (cut off) was defined as the minimum analyte concentration at which the colored line in the analytical zone could be observed visually. Instrumental LOD was defined as the analyte concentration at which the analytical zone coloration intensity exceeds by three times the standard deviation of the analytical zone coloration for zero samples (without FABP):

LOD = X_b1_ + 3S_b1_,

where X_b1_—the mean concentration of the blank (C (FABP) = 0 ng/mL), S_b1_—standard deviation of the blank [32].

## 3. Results

### 3.1. Common Test System

**Figure 2:**
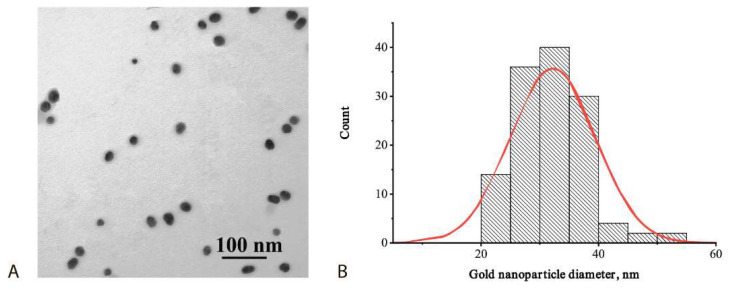
sGNPs: TEM image (**A**) and distribution of diameters (**B**).

At the first stage, a common immunochromatographic test system was realized and optimized. sGNPs were synthesized by the citrate technique and characterized by transmission electron microscopy—see Figure 2. The average diameter of the obtained nanoparticles was 32 ± 7 nm, which accords to generally accepted recommendations about the best size of GNPs for ICA [33].

The obtained sGNPs were conjugated with specific anti-FABP antibodies, F10. The optimal concentration of the antibodies for the conjugation was chosen based on the flocculation curve [27] and amounted to 10 µg/mL. Optimal conditions for the common ICA with sGNPs were chosen, taking into consideration our earlier development of ICA for FABP [26].

**Figure 3:**
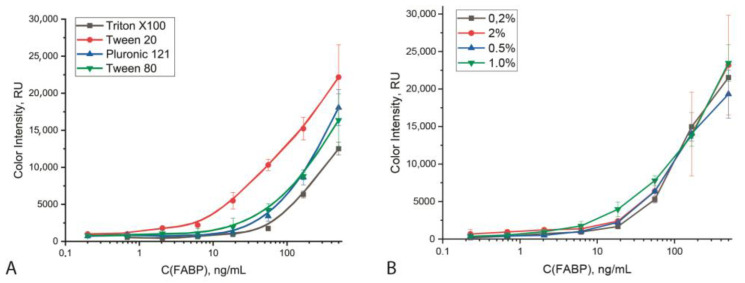
Dependence of the intensity of coloration of the analytical zone of the test strip on the nature of detergents (used at 1% concentration) (**A**) and the detergent concentration (for the case of Tween 20 use) (**B**) (*n* = 5).

Special consideration was given to the choice of detergent, a common compound in immunoassays that prevents non-specific interactions. Detergents of different chemical nature were tested: non-ionic surface-active compounds—Tween-20, Tween-80 and Triton X-100, which differ in the length of the polymer chain and, as a result, in solubility and micelle formation constant; and anionic surfactant Pluronic 121, with weak alkaline properties. Concentrations of the detergents varied from 0.2 to 5%. As can be seen from Figure 3, all tested detergents, except Tween-20, were characterized by a high background staining in ICA of FABP. The optimal concentration of Tween-20 was 0.2% (*v*/*v*), which makes it possible to reach an LOD equal to 11.2 ng/mL. Lower concentrations of the detergent impeded the movement of GNPs and increased the background coloration. On the other hand, an increase in the Tween-20 concentration led to higher LODs. Other parameters of the common ICA protocol, such as optical density of the applied GNP conjugate, concentration of antibodies applied to the analytical zone, etc. were chosen according to an earlier study [26].

**Figure 4:**
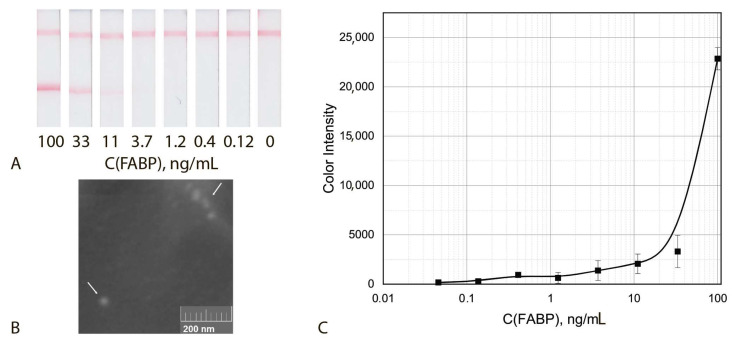
Common ICA protocol: Appearance of test strips (**A**), SEM image of the sGNPs bound in the analytical zone (**B**) and calibration curve for FABP detection in serum (**C**) (*n* = 5).

Finally, analytical parameters of the common ICA of FABP were determined under the optimized conditions. The given assay was characterized by cut off and LOD equal to 33 ng/mL and 11.2 ng/mL, respectively (Figure 4).

### 3.2. Test System with Changed Label

**Figure 5:**
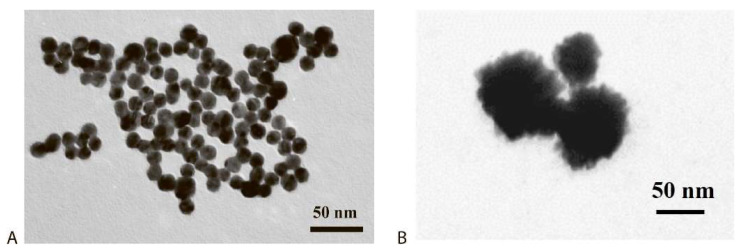
GNFs preparation: TEM images of the nuclei (**A**) and the final GNFs (**B**).

The advantages of GNFs are the larger size and significantly more developed surface of nanoparticles due to their complex structures (tips). These factors contribute to the immobilization of a larger amount of antibodies for more efficient antigen interaction, which reduces the detection limit of high molecular weight antigens. The benefits of GNFs are determined by their developed surface, which provides increased binding of antibodies and more efficient interaction with the target antigen, as specified in [16]. To use these alternate labels in our studies, the GNFs were synthesized using small sGNPs (nuclei) with average diameters of 10 ± 4 nm, further growing up to average diameters of 78 ± 4 nm (Figure 5). The obtained nanoparticles were used for the synthesis of conjugates with antibodies.

For the ICA using GNFs, the optimal concentration of antibodies in the conjugate with GNFs, the concentration of specific antibodies in the analytical zone of the test strip, and the concentration of the detecting conjugate were selected.

The optimal concentration of the F10/FABP antibodies for their conjugation with GNFs was 10 μg/mL, which coincides with the data for sGNPs. Comparison of the conjugates with different antibody loadings demonstrated that the maximum level of the analytical zone coloration was achieved with the maximum amount of antibodies used for the conjugate synthesis—20 μg/mL. However, the minimum cut off was achieved at 10 µg/mL; it was equal to 1.2 ng/mL.

**Figure 6:**
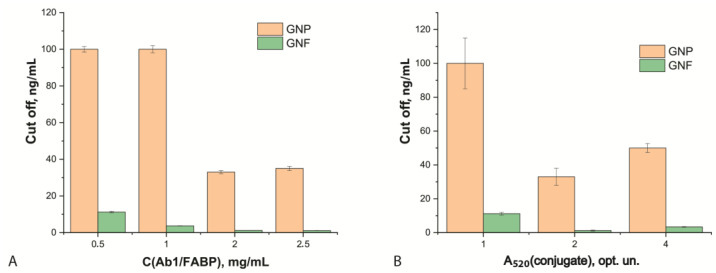
Choice of ICA conditions for test systems with GNPs and GNFs. Dependence of cut off values on the concentration of F5/FABP applied in the analytical zone (**A**) and on the optical density of the used antibody-nanoparticles conjugates solutions (**B**) (*n* = 5).

The optimal concentration of antibodies in the analytical zone of the test strip both for GNFs and sGNPs was 2 mg/mL, at which, the minimum cut off values were achieved (Figure 6A). The optimal optical density (A_520_) of the conjugate of GNFs and sGNPs was 2 opt. units; its use provided the minimum cut off (Figure 6B). Cut off was defined as the minimum analyte concentration at which the colored line in the analytical zone could be observed visually.

**Figure 7:**
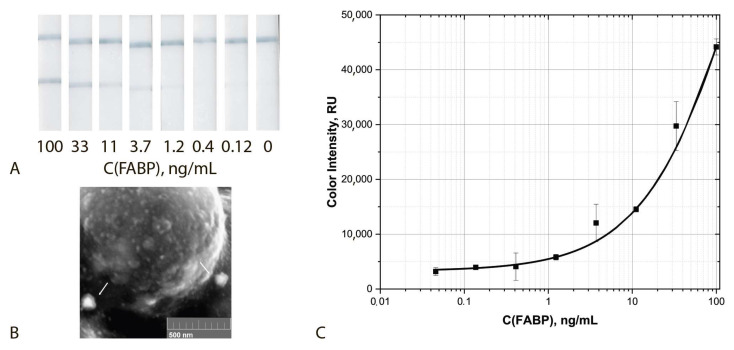
ICA with the use of GNFs: Appearance of test strips (**A**), SEM image of the GNFs bound in the analytical zone (**B**) and calibration curve for FABP detection in serum (**C**) (*n* = 5).

Under the chosen conditions, the test system based on a single GNF-F10 conjugate has a cut off and LOD of 1.2 ng/mL and 0.4 ng/mL, respectively (Figure 7). Thus, **replacing sGNPs with GNFs led to la owering of the LOD by a factor of 28 times (LOD) and 27 times (cut off).**

### 3.3. Test System with Nanoparticles Aggregation

Effectiveness of the aggregation method of signal amplification in lowering the LOD is determined by the introduction of multiple nanoparticle labels into the formed immune complexes [17,28]. Taking into account earlier studies of such assays conditions, we additionally optimized the choice of carrier protein for biotinylation and the ratio of the three conjugates used, namely, the detecting sGNP–IgG–biotin conjugate and the two enhancing sGNP–Stp and sGNP–biotin conjugates.

As an additional biotinylated component, bovine serum albumin and soybean trypsin inhibitor were considered as two proteins not involved in specific interactions. Both proteins were biotinylated by standard protocol and conjugated with sGNPs. 

The analytical parameters of ICA reached for these two cases are summarized in Table 1. As can be seen, a lower LOD was achieved when BSA was used as a supplemental biotin carrier. This difference may be explained by the fact that the molecular weight of STI is three times lower than the mass of BSA. The amount of additional biotin is less and, as a result, the formation of biotin–streptavidin links in the course of the ICA is less efficient [34].

The use of three aggregating conjugates led to an increase in the background signal and uneven movement of the conjugates along the working membrane while maintaining the analytical characteristics of the test system. In this connection, individually adsorbed GNP-Stp conjugate and a mixture of GNP-BSA-biotin and GNP-F10-biotin conjugates were used. This combination minimized the background coloration of the test strip.

**Figure 8:**
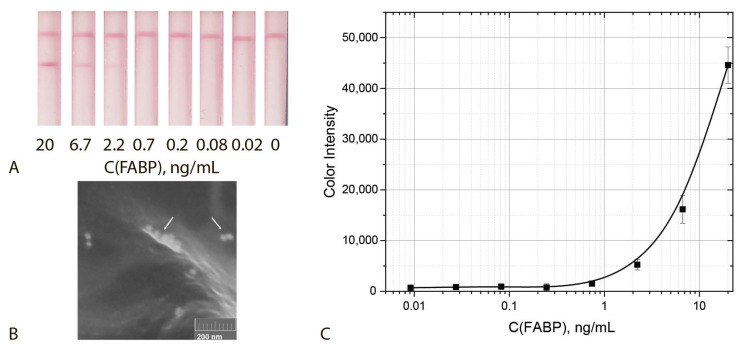
ICA with the use of GNPs aggregation: Appearance of test strips (**A**), SEM image of the sGNPs aggregates bound in the analytical zone (**B**) and calibration curve for FABP detection in serum (**C**) (*n* = 5).

The aggregating ICA protocol under the chosen optimal conditions was characterized by a cut off and LOD equal to 2.2 ng/mL and 0.7 ng/mL, respectively (Figure 8). Thus, **the use of the aggregation system based on the sGNPs allowed for a reduction of the LOD by a factor of 15 times (LOD) and 14 times (cut off).**

**Figure 9:**
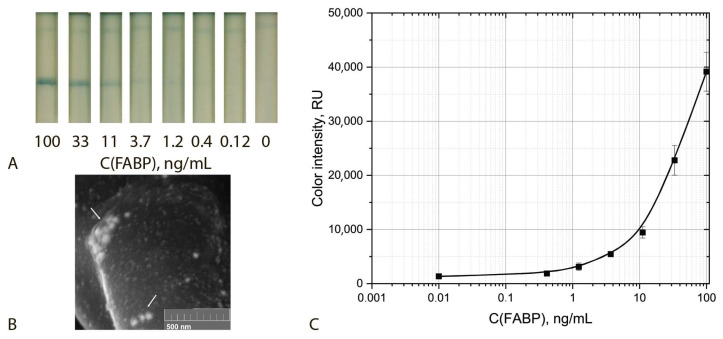
ICA with the use of GNFs aggregation: Appearance of test strips (**A**), SEM image of the sGNFs aggregates bound in the analytical zone (**B**) and calibration curve for FABP detection in serum (**C**) (*n* = 5).

Another completion of the aggregation system included a detecting GNF–F10–biotin conjugate and two amplifying sGNP–Stp and GNF–biotin conjugates. This ICA was characterized by a cut off and LOD equal to 1.2 ng/mL and 0.3 ng/mL, respectively (Figure 9). The lower reached values were in accordance with more sensitive assays using GNFs as compared with GNPs, as described above. Thus, **the use of an aggregation system based on GNFs allowed for a reduction of the LOD by a factor of more then 100 times (LOD) and 33 times (cut off).**

### 3.4. Triple Enhanced Test System

**Figure 10:**
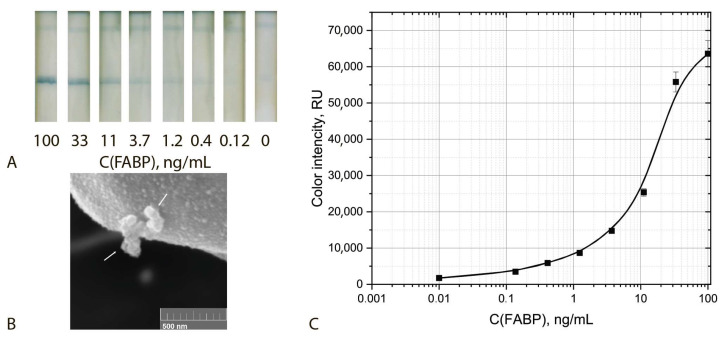
ICA with triple enhancing approaches: appearance of test strips (**A**), SEM image of the sGNFs aggregates bound in the analytical zone (**B**) and calibration curve for FABP detection in serum (**C**) (*n* = 5).

Finally, the aggregation ICA protocol with GNFs as the most efficient double combination was integrated with post-assay growth of gold nanoparticles by reduction of gold salt. This additional technique increases the coloration of the label bound to the formed immune complex, and thus, also causes a lowering of the ICA LOD, as demonstrated in its earlier application for various analytes [35,36]. The post-assay growth of nanoparticles was confirmed by comparison of SEM data comparing size of the labels attached to immune complexes formed at the test strip—see Figure 4B, Figure 7B, Figure 8B, Figure 9B and Figure 10B for comparison.

Thus, the implemented assay included aggregation of three kinds of functionalized nanoparticles, namely, detecting GNF–F10–biotin conjugate and two enhancing sGNP–Stp and GNF–biotin conjugates and post-assay enhancement by using H_2_O_2_ and HAuCl_4_. The incubation time of the test strip with the growing solution varied from 1 to 5 min. The optimal time was 2 min, which made it possible to obtain well-reproducible results. Increasing the incubation time leads to an increase in non-specific interaction and the formation of a high background signal. The assay implemented under optimal conditions was characterized by cut off and LOD values equal to 0.4 ng/mL and 0.05 ng/mL, respectively (Figure 10). Thus, **the integration of three enhancing approaches made it possible to reduce the LOD bya factor of 220 times (LOD) and 80 times (cut off)**. The size of the aggregates in the analytical zone increased by a factor of 3–5 times (see SEM images at the Figure 9B and Figure 10).

To avoid non-correct optimizations, the presented testing of ICA with different combinations of enhancing approaches were implemented using the target analyte (FABP) in a target matrix (serum). Finally, the practical applicability of the proposed triple enhancing assay was additionally evaluated by quantitative characterization of FABP recovery in spiked samples. A high rate of FABP recovery was shown, reaching 98 ± 2% (C(FABP) = 33 ng/mL). For all developed test systems, reproducibility was evaluated by the coefficient of variation in the range from 8 to 12% in the working ranges.

## 4. Discussion

Table 2 summarizes the reached analytical parameters of ICA with different combinations of enhancing approaches. The reached improvements for the combined three approaches are in accordance with the earlier presented data about their application in the detection of different analytes.

Gold nanoparticles are the most widely used labels in LFIA due to the simplicity of their preparation and the variation of properties, the possibility of effective functionalization and the low detectable concentrations caused by plasmonic properties. Replacing spherical nanoparticles with alternative gold nanoparticles, anisotropic or non-oriented, but with a developed surface, provides for a lowering of the ICA LOD [37,38]. Thus, recent studies have shown advantages of flower-like gold nanoparticles with a developed surface in the forming of wavy or sharp (tips) protrusions [39,40,41,42].

A promising method for lowering the ICA LOD is to increase the amount of colored marker in the assay area of the test strip, which allows for an increase of the intensity of the colorimetric signal. The formation of aggregates or networks of functionalized small nanoparticles makes it possible to avoid non-specific binding or difficulties in the migration of particles along the membrane [8,43]. For the formation of aggregates, intermolecular interactions such as biotin–streptavidin, antibody–antispecies antibody and antibody–antigen were successfully used [44,45]. This approach is characterized by easy implementation, the elimination of additional washing steps or the use of “wet” chemistry.

The growth of nanoparticles by reducing gold or silver salts on their surface can significantly lower the LOD [46]. Various silver recovery protocols have been published, ranging from 3-fold to 100-fold sensitization, but these treatments use strong non-specific staining and cannot be used for chloride preparations [36,47,48]. The reduction of gold salt on the GNP surface is preferable because it eliminates these drawbacks [49].

In contrast to the considered individual application of different enhancing approaches, the presented study demonstrates that their integration in the same test system leads to multiple lowerings of the detection limit and a final improvement of the analytical parameters by more than two orders of magnitude, thereby significantly extending the possibility for on-site immunochromatographic control of various analytes. 

The data regarding the existing developments of ICA for FABP collected in Table 3 demonstrate the high competitive potential of the developed sensitive assay. Note that the proposed improvements are often based on alternate detection techniques, such as fluorimetry, which need additional instrumental ensuring. In contrast, all approaches used in the work remain within the framework of optical registration of results with the possibility of instrumentless testing.

## 5. Conclusions

The work proposes the integration of three approaches for lowering the LOD of immunochromatographic detection of fatty acid binding protein (FABP), namely: (1) replacement of spherical gold nanoparticles with gold nanoflowers (20 times); (2) formation of aggregates in the analytical zone (15 times); and (3) growth of nanoparticles by gold salt reduction (3 times). It is shown that the combination of three methods integrates the impacts of the individual amplification factors into lowering the limit of detection. The final limit of detection of FABP was 0.05 ng/mL, which is 220 times lower than the common test system.

The developed test system, in addition to its undeniable advantages, has disadvantages. The main advantage of the proposed approach lies in the very low detection limit of the analysis, which exceeds each of the methods used separately. Combining the three methods slightly increases the assay time, which does not affect its rapidity.

Its implementation requires the use of additional reagents, which is an additional stage that increases the analysis time by 1 min. However, the introduced changes slightly affect the total analysis time while significantly improving the analytical performance. The transfer of the proposed test system into a dry format by finding the best protocols of extra reactants to be applied on an additional membrane seems to be a reasonable task for further investigations to overcome this limitation.

The triple enhanced test system meets the requirements of AGREE (Analytical GREEnness Metric Approach) [54], as well as immunochromatography in general. ICA uses the smallest sample volume to directly quantify the analyte. The proposed modification of the assay protocol is not associated with the involvement of new toxic reactants or increased pollution in the course of the assay implementation.

## Figures and Tables

**Table 1:** LODs for aggregation ICA using two protein carriers.

Biotinylated Protein	LOD, ng/mL	Cut off, ng/mL
Bovine serum albumin	0.7	2.3
Soybean trypsin inhibitor	2	11.2

**Table 2:** Integrated characterization of the developed ICA of FABP by their LOD and cut off values.

Labels of the Formed Immune Complexes	LOD, ng/mL	Cut off, ng/mL
sGNP–IgG	11.2	33
GNF–IgG	0.4	1.2
sGNP–IgG–biotin—sGNP–Stp—sGNP–biotin	0.7	2.3
GNF–IgG–biotin—sGNP–Stp—GNF–biotin	0.1	1
GNF–IgG–biotin—sGNP–Stp—GNF–biotin + reduced gold salt	0.05	0.4

**Table 3:** Comparative characteristics.

Marker	Detection Technique	LOD, ng/mL	Ref.
Gold nanoparticles	Photometry	3.8	[26]
Gold nanoparticles	Photometry	1.5	[50]
ZrMOF@CdTe quantum dots	Fluorimetry	1	[51]
CdTe quantum dots	Fluorimetry	0.221	[52]
Fluorescence composite nanostructures	Fluorimetry	0.21	[53]
Combined gold markers with catalytic enhancement	Photometry	0.05	This work

## Data Availability

The data that support the findings of this study are available from the corresponding author upon request.
